# A best-fit solution: transforming an NHS Library and Knowledge Service in readiness for a new hospital building without a traditional library space

**DOI:** 10.5195/jmla.2021.1167

**Published:** 2021-07-01

**Authors:** Rebecca Jean Scott

**Affiliations:** 1 beckyjscott@icloud.com, Formerly Library and Knowledge Services Manager at Royal Papworth Hospital, Currently Information Manager for the School of Health and Social Work at the University of Hertfordshire

**Keywords:** transformation, embedded librarianship, innovation, project management

## Abstract

**Background:**

Over a decade ago, the Hill report argued that a shift in vision was required to change the perception of National Health Service (NHS) Library and Knowledge Services (LKS) in England from “book repositories” to essential services that underpin clinical decision-making by patients, carers, and health care professionals. Health Education England's *Knowledge for Healthcare: A Development Framework for Library and Knowledge Services in England 2015–2020* advocates embedding librarians within clinical and management teams in order to provide access to high-quality evidence at the point of need.

**Case Presentation:**

In April 2019, Royal Papworth Hospital relocated twelve miles from its historic village location in Papworth Everard to its new state-of-the-art hospital on the Cambridge Biomedical Campus. The design for this new hospital did not accommodate a traditional library space and therefore necessitated a transformation of the LKS. The organization opted to embed the LKS staff into the clinical setting and relegate 80% of the print collection to off-site storage. This project and its associated steps are presented as an example of health care library transformation.

**Conclusion:**

Embedding the LKS team in the clinical setting, engaging in proactive outreach activity, and improving our marketing led to a 44% increase in literature searches requested compared to the same eleven-month period in the previous year. A 40% decrease in our print book loans indicates additional barriers to using a click-and-collect service and the need for greater investment in our e-book provision. However, early outcomes for our best-fit service transformation are positive. Having an open, dual mindset has enabled the service to embrace change and maximize emerging opportunities to collaborate with clinical staff on new projects.

## BACKGROUND

Over a decade ago, the Hill report argued that a shift in vision was required to change the perception of National Health Service (NHS) Library and Knowledge Services in England from “book repositories” to essential services that underpin clinical decision-making [1]. Integrating Library and Knowledge Services (LKS) staff into the clinical setting is central to Health Education England's vision for NHS LKS in England [2]. *Knowledge for Healthcare: A Development Framework for Library and Knowledge Services in England 2015–2020* outlines the strategic framework to transform LKS to meet the needs of health care organizations for the twenty-first century. The framework advocates the view that LKS must underpin clinical and management decision-making and outlines embedding librarians within clinical teams as a strategic priority.

Embedded librarians are co-located with their users and are focused on supporting users' needs within their environment or workplace [3]. Clinical librarians provide high-quality information directly to clinicians to inform decisions around patient care, safety, and service provision [4]. These individuals are considered part of the clinical team and are integrated into ward rounds, case review, and audit meetings [4]. Clinical librarianship in the United States has a long-established history of librarians supporting clinicians at the point of care [5]. In recent years in the East of England, NHS libraries have increased the number of staff working in clinical librarian roles. The types of activities they undertake are diverse: supporting systematic reviews, attending ward rounds and multidisciplinary team meetings, and running journal clubs [6].

During the period 2015 to 2017, there was a successful clinical librarian program at Royal Papworth Hospital, with three qualified librarians supporting systematic reviews, delivering training in critical appraisal and evidence searching skills, and attending corporate and clinical meetings. However, after two members of the team left the organization, the clinical librarian program was abandoned. In January 2018, the organization appointed a new LKS manager. The hospital was due to relocate in September 2018 to a new site, and the hospital design did not accommodate a traditional library space. The organization tasked the LKS manager with identifying a solution for the operation of the LKS. Various options were explored and ruled out; for example, a service-level agreement with a higher education institution was initially pursued but was deemed unfeasible due to budget constraints. The option to share space in a different NHS organization was briefly considered but deemed unsuitable because this would potentially increase barriers to accessing LKS services.

The vision in *Knowledge for Healthcare* for the future of NHS LKS in England was to embed librarians within the clinical setting; therefore, the LKS manager submitted a proposal to the executive team to situate the LKS team within the clinical administration area and adopt a hybrid service model. The LKS manager did not propose a fully virtual service, as the existing print collection at Royal Papworth was highly specialized because the hospital is a specialist acute center treating patients with heart and lung diseases. Furthermore, the high cost of replacing the print collection with an equivalent e-collection was not practical due to the lack of funding available for the project and the high cost of electronic resources. The organization approved the recommended proposal for the embedded hybrid model in early 2019.

This case report will describe the project to implement the best-fit solution of the embedded hybrid LKS for Royal Papworth Hospital and its associated outcomes eleven months later. This case is presented as an example of health care library transformation.

## CASE PRESENTATION

The initial deadline for the hospital move was September 2018; however, construction issues delayed the move to April 2019. During this sixteen-month period, after exploring various proposals, the organization opted to embed LKS staff into the clinical setting and relegate 80% of the print collection to off-site storage. Study space had been designed as fourteen individual booths located in office areas of the hospital, and each was equipped with a desk, computer, and telephone. Hot desking facilitated staff access to computers. Hot desking and touchdown bases are increasingly common in UK workplaces. This flexible approach to workspace means staff have no allocated personal deskspace and use any designated hot desk to access a PC or a touchdown base to connect their laptop to the network [7].

The project used a traditional four-stage approach to project management: start, plan, implement, and close [8]. The project aimed to transform the LKS service for the new Royal Papworth Hospital into an embedded hybrid model.

### Stage 1: Start

In the very early phase of the project (June 2018), the LKS manager surveyed library users to identify what they perceived to be the most important aspects of the existing service. Sixty-six percent of respondents cited the physical print collection as most important to them. This response underpinned the decision to propose a hybrid model that retained the print collection. The LKS manager presented a paper listing five potential options for the LKS to the executive team for review:

- Service level agreement with higher education institution (HEI)
- Shared service model between HEI and Royal Papworth
- Fully virtual service with minimal staffing model
- Fully virtual service delivered remotely with full staffing model
- Hybrid embedded service model

The executive team assessed each option against how effectively it met quality assurance criteria and organization's needs [9]. The executive team requested an amendment to the paper, reducing the potential options to three and identifying the hybrid model as the preferred option. The organization formally approved the proposal in February 2019, and the LKS team reached their first milestone. While awaiting final formal approval, an operational procedure document was created and submitted for review to the broader new hospital project team.

### Stage 2: Plan

Departmental planning for the transformation involved the following elements: collection management, operational management, space, staffing, and users.

### Collection management

In September 2018, a phased weeding of the print collection commenced using the existing collection development policy. The criteria for withdrawal were as follows:

- Items that had a newer edition in our electronic or print collection
- Items that had not been loaned in the previous ten years
- Items that had not been loaned in the last five years and that were available for interlibrary loan in our regional consortium
- Items in poor condition
- Items that were out of date for clinical practice or not in line with current policy

The LKS team completed weeding in December 2018.

The LKS manager used library management system (LMS) reports to identify the most frequently used print books in the collection and then applied the 80/20 rule [10]. She identified the most popular 20% of the print collection (~350 items) to be stored in a small basement room on-site at the new hospital. The remaining 80% of items were relegated to an off-site storage facility that was already approved for storing and retrieving hospital records. Books could be recalled from the store and delivered by courier to the hospital within forty-eight hours.

The team updated the new location status of all print items in the LMS. The relegated items were catalogued in the hospital records online platform and boxed for dispatch to the storage facility a week before the hospital move.

The LKS manager also had to find a new home for a historic collection of print materials from an eminent deceased cardiologist at Royal Papworth. With support from the immediate past president of the British Cardiovascular Society, the LKS manager secured a permanent donation of the materials, and the collection was transferred in January 2019.

The LKS provides access to a broad range of e-journals. Health Education England funds a national collection of e-journals, and the LKS supplements this with local subscriptions to meet the organization's needs as a specialist cardiothoracic center. No subscriptions to print journals were in place. Data collected in the previous five years demonstrated that the existing historic print journals had minimal use. The LKS manager decided to preserve the book collection over the print journal collection because the priority for the organization is current clinical practice rather than the history of cardiothoracic medicine. Therefore we offered our historic print journals to other libraries in the East of England region, and these were boxed and sent by courier to the receiving libraries in March 2019.

The LMS's existing reservation feature was utilized to enable a “click-and-collect” method of print book requests. We created a user guide to offer step-by-step instructions on how to order items in preparation for the new ways of working.

### Space

With support from the wider hospital project team, one allocated desk was identified for the LKS team on the first-floor clinical administration area. The clinical administration area consisted of three interconnected open plan offices. Each office hosts a different specialty: cardiology, thoracic, and transplant. The LKS desk was situated in the center office for thoracic clinical staff and was adjacent to the desks for respiratory consultants and the lung infection team and would provide a consistent enquiry point for face-to-face user enquiries. The other members of LKS team would use a combination of hot desking, remote working, and touchdown bases. Six of the hospital study booths were also located in the same office area, as well as several meeting rooms that were bookable for group library training sessions. Following desk allocation, the LKS manager visited the new hospital site and identified a nearby area appropriate for a book returns unit and a small trolley for users to collect their items.

### Staffing

**Figure 1:**
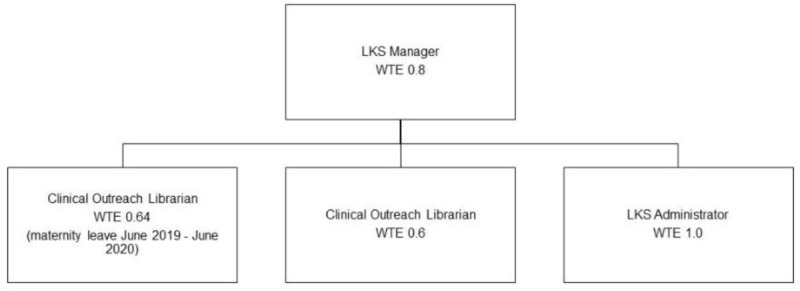
LKS departmental structure

The LKS manager recruited two new staff to the team during the move to the new site. An additional part-time clinical outreach librarian joined the team in April 2019 and a full-time LKS administrator in May 2019. The departmental structure is presented in Figure 1.

### Users and communication

The LKS team emailed users an outline of the new arrangements for library services in March 2019 and notified staff through the organization's move briefing. The LKS manager sent all users an email reminder of their username and password in April 2019 and the user-guide for the user interface of the LMS so that they were operationally ready to use the “click-and-collect” service. The LKS team extended loan periods to cover the move period and allow a grace period for return of items after the move.

The LKS manager updated the content of the library pages on the hospital website to ensure that the service had a strong digital presence and that users could easily navigate the services available. We developed online forms to give users an easy way to request services, such as literature searches and training bookings. We added a frequently asked questions page and a set of directions with pictures on how to find the enquiry desk to the website.

### Stage 3: Implementation

The hospital relocation spanned twelve days, and the LKS team transferred to their new home on day eight along with the items to house in the basement. The LKS was fully operational on day eleven.

The implementation of the project required adaptation to the new hospital environment. Without the clear demarcation of a traditional library space, the greatest challenge was communicating a clear message to our users about how to access library services.

### Outreach and marketing

The clinical outreach librarian (COL) developed a weekly ward visits schedule to enable staff to easily access services and troubleshoot common issues as they arose. The COL secured support for this outreach from ward sisters in June 2019. In England, ward sisters are nursing leaders who have responsibility for managing the ward and the nursing staff on a shift-to-shift basis [11]. From July 2019 to March 2020, the COL conducted 111 ward visits, resulting in forty-two new user registrations and 193 enquiries (see appendix for details of enquiries by month). The most commonly asked questions were:

- How do I join the library? (18%)
- Where can I find library services? (15%)
- How do I order a print book? (11%)

The LKS administrator also visited the critical care education room weekly, which led to the COL delivering a regular thirty-minute workshop as part of their clinical study days on the topic of evidence-based practice.

The COL secured invitations to four clinical and therapeutic team meetings to build relationships and enable access to services. In addition, the COL held popup stalls in the hospital atrium to promote the service and increase visibility. Often these were tied to events, for example, World Heart Day and Libraries Week. These events generated leads for future projects, including a joint initiative between the LKS and occupational therapy to loan audiobooks to patients awaiting cardiothoracic surgery and COL involvement in a pharmacy research café.

The LKS administrator spearheaded improving marketing by creating a monthly newsletter, targeting welcome emails to job profiles for new staff, and rebranding some of our training sessions. We frequently updated the library pages of the hospital website as we responded to user queries about available services.

### Stage 4: Close

The project was considered closed at the end of the financial year (March 2020). At this point, eleven months after relocating, both users and LKS staff were familiar with the new ways of working. Furthermore, in March 2020, the COVID-19 pandemic impacted both the country and the organization [12, 13]. The LKS team pivoted to delivering services online and supporting the organization by fulfilling other tasks in addition to their normal roles. The initial intention had been to survey users at the twelve-month endpoint; however, with the impact of the COVID-19 pandemic, this was not feasible.

However, the LKS team sought user feedback at three- and six-month increments. At three months, users expressed frustration in not being able to browse the collection. This response was also a frequent topic of conversation between users and LKS staff. Our catalog (a regional consortium LMS) did not have sophisticated search functionality. We adapted by updating catalog records to a more granular level, adding chapter titles to help users retrieve more useful results in their searches. At six months, user concerns shifted toward challenges in accessing e-journal articles and were no longer solely focused on the method of service delivery. This feedback could suggest that our users had begun to move through Kubler-Ross's change cycle to a state of acceptance of our embedded hybrid model [14].

The new service model outcomes included an increase in requests for literature searches by 44% compared to the same eleven-month period in the previous year. We anticipated that print book loans would decline due to the lack of browsing and the need to use a “click-and-collect” service; this materialized with a 40% decrease in loans compared to the same eleven-month period in the previous year. Usage of e-books did not increase, which indicates that users did not simply migrate their use of our resources to a different format. We did not significantly increase our e-book provision during the pre-move period and instead relied heavily on our existing collection through Clinical Key [15], which offers a good range of clinical textbooks, although nursing-specific content is limited. Nursing staff were the predominant print book users, and thus it is likely that we were not meeting their information needs. During the implementation phase, we opted for a method of patron-driven acquisition and purchased new titles as the need arose on a separate e-book platform. Going forward, Health Education England provided additional funding for e-books and procured a new shared platform for LKS in England, and we have secured local funds to add more titles.

## DISCUSSION

There are many examples in the professional literature of library service transformation. In the United States, Piedmont Atlanta Hospital transitioned from a physical library to a virtual one in 2012, a decision influenced by several factors, including changes in user behavior, budget cuts, and a reduction in staffing [16]. The University of Florida's Health Science Center Library chose to close the reference desk and moved to a method of “house calls,” creating roles such as clinical research librarian [17]. This role focused on providing services at the point of need through attending ward rounds and relevant clinical committees. The authors recommend a “flexible, fearless mindset” to seize the opportunities that change can bring.

A dual mindset is also considered vital for successful start-up businesses [18]. On the one hand, the team's focus must be on the core mission. On the other hand, there needs to be agility and a readiness to diverge from the path to meet new needs as they arrive [18]. In the case of the embedded Royal Papworth LKS, the core mission is to support the best possible patient care through delivering evidence at the point of need. In the eleven months after relocation, there was a clear increase in the number of evidence searches requested. Additionally, being closely situated with clinical staff enabled increased opportunities for networking. There were many emerging opportunities to embrace, which required an agile, open mindset: loaning audiobooks to patients, supporting the pharmacy research cafe, staff working on professional doctorates, and multi-center rapid reviews. Tennant et al.'s recommendations from their service transformation included being “open to change, even if the change appears to be potentially detrimental. If change is inevitable, develop strategies to identify and seize potential benefits” [17]. This recommendation is fully borne out by our own experiences of a best-fit library service transformation. The freedom to operate without the need to maintain a physical presence in a traditional library resulted in more time to collaborate with clinical staff on different projects.

In England, prior to the COVID-19 pandemic, a small number of library services had begun to operate without a traditional space. Library and Information Services at The Francis Crick Institute, a fundamental biology research institution, opened in 2016 and operated without a physical library space to facilitate science's collaborative nature [19]. In 2018, a new digital LKS was formed for ambulance staff delivering four service areas (current awareness, literature searches, reading lists, and article supply) via a website [20]. As a result of the COVID-19 pandemic, a greater number of health care libraries shifted to deliver their services virtually through platforms such as Microsoft Teams [21]. It seems unlikely that health libraries will entirely revert to the traditional model.

The LKS manager requested feedback from senior leaders in the organization about the perceived outcomes of the transformation, which was overwhelmingly positive. The research and development manager commented that the LKS is now “a more integrated service with the clinical teams as you are co-located. I think it has been a phenomenal success” [V. Hughes, email, Dec 22, 2020]. Furthermore, the acting chief nurse elaborated on this from the organization's perspective, stating “it will enhance sustainability of the service and longevity, particularly in a rapidly changing digital world. It has meant the service can be more agile and responsive” [I. Graham, email, Dec 22, 2020]. During the eleven-month period, staff in senior roles started utilizing our expertise where previously they had not, and this reach continues to increase (e.g., assistant director of education, non-executive director, surgery clinical director). This indicates further the successful outcome of our service transformation.

It is important to acknowledge that service transformation is not without significant challenges. First, there is a need to upskill the LKS workforce in the UK to successfully perform embedded and clinical librarian roles [22]. Second, some traditional services may need to cease for small teams to have the ability to deliver on collaborative projects. As Tennant et al. explain, “No library can do it all” [17]. We reduced our acquisitions of print materials significantly after the move and therefore saved time through reduction in physical processing, labeling, and cataloging of items. The LKS manager decided to suspend promotion of systematic review support because one experienced team member went on maternity leave, and it is a highly labor-intensive service. Our training program was very limited in the first three months after the move. We prepared for this by prerecording videos of our popular workshops (academic writing, writing for publication, citing, and referencing) so that staff could continue to access them while we adjusted to new ways of working. Critical appraisal training was not offered for the eleven-month duration. Although our evidence searches increased significantly during the project period, our print book collection usage declined. This decline could have been influenced by several barriers to access: closed shelves, limited search functionality on the LMS user interface, users' lack of digital skills, and clinicians' lack of free time for education and continuous professional development. Users who attend our training sessions often do so on their day off. Thus there is a need to re-engineer services to meet staff needs who struggle to be released from their clinical duties and advocate for protecting time to attend learning and professional development opportunities. A blended approach to online training with synchronous and asynchronous sessions could facilitate better access for staff who wish to attend training in their own time. Face-to-face training could be embedded within clinical team meetings to facilitate attendance, and this could potentially foster an evidence-based culture within teams.

The future direction of health care libraries will perhaps see more hybrid models with staff rotating between on-site outreach and ward-based support and remote working service delivery. Development opportunities for LKS staff to perform new roles will need to be developed. These could be shadowing opportunities where LKS staff are able to work alongside more experienced clinical librarians in other hospitals. Also, an international online mentoring scheme could be implemented with colleagues from the United States who have a long-established culture of clinical librarianship and could offer the benefit of their expertise.

In conclusion, the project's early outcomes to transform the Royal Papworth LKS to an embedded service model are positive. A notable increase in evidence search requests indicates more awareness of and greater access to the evidence search service. The decrease in print book loans indicates a need to support staff in accessing the “click-and-collect” service and to further improve search functionality. There is a need for continued investment in e-book provision to better meet the needs of our staff. The numerous opportunities to participate in projects across the organization suggest that our best-fit solution has successfully created a service that maximizes the benefits of delivering a library service for a new hospital without a traditional library space. The open, dual mindset has enabled a successful health care library service transformation.

## Data Availability

Data associated with this article are available in the Open Science Framework at <https://osf.io/gm3j5>.
