# Supplementary material for: A best-fit solution: transforming an NHS Library and Knowledge Service in readiness for a new hospital building without a traditional library space
Source: J Med Libr Assoc. 2021 Jul 1;109(3):483–9. doi: 10.5195/jmla.2021.1167 (PMC8485953; doi:10.5195/jmla.2021.1167)
Supplement: Supplementary file 1 — Appendix [file jmla-109-3-483-s01.docx]

**Appendix**

| **Type of enquiry** | **Jul-19** | **Aug-19** | **Sep-19** | **Oct-19** | **Nov-19** | **Dec-19** | **Jan-20** | **Feb-20** | **Mar-20** | **Total** |
| --- | --- | --- | --- | --- | --- | --- | --- | --- | --- | --- |
| How do I join the library? | 16 | 9 | 5 | 1 | 1 | 1 | 1 | 0 | 1 | **35** |
| How do I order a print book? | 5 | 9 | 2 | 3 | 0 | 1 | 2 | 0 | 0 | **22** |
| Questions related to OpenAthens | 14 | 3 | 2 | 2 | 0 | 0 | 0 | 0 | 0 | **21** |
| What's my PIN/ID? | 9 | 5 | 6 | 0 | 0 | 0 | 1 | 0 | 0 | **21** |
| Where can I find Library Services? | 7 | 5 | 6 | 6 | 0 | 1 | 3 | 0 | 1 | **29** |
| I don't know if I have a library account | 0 | 2 | 4 | 0 | 0 | 0 | 0 | 0 | 0 | **6** |
| I'm interested in training | 1 | 5 | 3 | 0 | 0 | 0 | 0 | 0 | 0 | **9** |
| Do you have (name of book)? | 1 | 3 | 5 | 1 | 0 | 0 | 2 | 0 | 1 | **13** |
| Can I browse books? | 0 | 1 | 2 | 0 | 2 | 1 | 0 | 0 | 0 | **6** |
| How do I renew my book(s)? | 1 | 1 | 0 | 0 | 1 | 0 | 0 | 0 | 0 | **3** |
| Do we subscribe to (name of journal)? | 2 | 0 | 0 | 0 | 0 | 0 | 0 | 0 | 0 | **2** |
| How can I get a journal article? | 1 | 0 | 0 | 0 | 0 | 0 | 0 | 0 | 0 | **1** |
| Is there study space? | 2 | 0 | 1 | 0 | 0 | 0 | 0 | 0 | 0 | **3** |
| Do we have a library? | 0 | 0 | 1 | 0 | 2 | 0 | 1 | 1 | 1 | **6** |
| Do you take old books? | 0 | 0 | 1 | 0 | 0 | 0 | 0 | 0 | 0 | **1** |
| Questions related to UpToDate | 0 | 0 | 1 | 0 | 0 | 0 | 0 | 0 | 0 | **1** |
| Can I have help with a literature search? | 0 | 0 | 1 | 0 | 0 | 0 | 0 | 0 | 0 | **1** |
| How do I return a book? | 0 | 0 | 0 | 0 | 1 | 0 | 0 | 0 | 0 | **1** |
| Other specific enquiries | 0 | 0 | 3 | 2 | 0 | 0 | 2 | 4 | 1 | **12** |
| **Total** | **59** | **43** | **43** | **15** | **7** | **4** | **12** | **5** | **5** | **193** |
